# Identifying the research, advocacy, policy and implementation needs for the prevention and management of respiratory syncytial virus lower respiratory tract infection in low- and middle-income countries

**DOI:** 10.3389/fped.2022.1033125

**Published:** 2022-11-09

**Authors:** Xavier Carbonell-Estrany, Eric A. F Simões, Louis J Bont, Angela Gentile, Nusrat Homaira, Marcelo Comerlato Scotta, Renato T Stein, Juan P Torres, Jarju Sheikh, Shobha Broor, Najwa Khuri-Bulos, D James Nokes, Patrick K Munywoki, Quique Bassat, Arun K Sharma, Sudha Basnet, Maria Garba, Joanne De Jesus-Cornejo, Socorro P Lupisan, Marta C Nunes, Maduja Divarathna, John R Fullarton, Barry S Rodgers-Gray, Ian Keary, Mark Donald C Reñosa, Charl Verwey, David P Moore, Faseeha Noordeen, Sushil Kabra, Marynéa Silva do Vale, Rolando Paternina-De La Ossa, Cristina Mariño, Josep Figueras-Aloy, Leonard Krilov, Eitan Berezin, Heather J Zar, Krishna Paudel, Marco Aurelio Palazzi Safadi, Ghassan Dbaibo, Imane Jroundi, Runa Jha, Rukshan A. M Rafeek, Rossiclei de Souza Pinheiro, Marianne Bracht, Rohitha Muthugala, Marcello Lanari, Federico Martinón-Torres, Ian Mitchell, Grace Irimu, Apsara Pandey, Anand Krishnan, Asuncion Mejias, Marcela Santos Corrêa da Costa, Shrijana Shrestha, Jeffrey M Pernica, Felipe Cotrim de Carvalho, Rose E Jalango, Hafsat Ibrahim, Atana Ewa, Gabriela Ensinck, Rolando Ulloa-Gutierrez, Alexandre Lopes Miralha, Maria Florencia Lucion, Md Zakiul Hassan, Zubair Akhtar, Mohammad Abdul Aleem, Fahmida Chowdhury, Pablo Rojo, Charles Sande, Abednego Musau, Khalequ Zaman, Luiza Helena, Falleiros Arlant, Prakash Ghimire, April Price, Kalpana Upadhyay Subedi, Helena Brenes-Chacon, Doli Rani Goswami, Mohammed Ziaur Rahman, Mohammad Enayet Hossain, Mohammod Jobayer Chisti, Nestor E Vain, Audrey Lim, Aaron Chiu, Jesse Papenburg, Maria del Valle Juarez, Thamarasi Senaratne, Shiyamalee Arunasalam, Tor A Strand, Adaeze Ayuk, Olufemi Ogunrinde, Lohanna Valeska de Sousa Tavares, Comfort Garba, Bilkisu I Garba, Jeanette Dawa, Michelle Gordon, Eric Osoro, Charles N Agoti, Bryan Nyawanda, Mwanajuma Ngama, Collins Tabu, Joseph L Mathew, Andrew Cornacchia, Ganesh Kumar Rai, Amita Jain, Mateus Sfoggia Giongo, Bosco A Paes

**Affiliations:** ^1^ Neonatology Service, Hospital Clinic, Barcelona, Spain; ^2^ Department of Pediatrics, School of Medicine, University of Colorado, Aurora, CO, United States; ^3^ Department of Epidemiology, Center for Global Health, Colorado School of Public Health, University of Colorado, Aurora, CO, United States; ^4^ Laboratory of Translational Immunology and Department of Paediatrics, Wilhelmina Children's Hospital, University Medical Centre Utrecht, Utrecht, Netherlands; ^5^ Epidemiology Department, Austral University, Buenos Aires, Argentina; ^6^ Ricardo Gutiérrez Children Hospital, Buenos Aires, Argentina; ^7^ School of Women's and Children's Health, Faculty of Medicine, University of New South Wales, Sydney, NSW, Australia; ^8^ Respiratory Department, Sydney Children's Hospital, Randwick, NSW, Australia; ^9^ Pontificia Universidade Católica do Rio Grande do Sul (PUCRS), Porto Alegre, Brazil; ^10^ Hospital Moinhos de Vento, Porto Alegre, Brazil; ^11^ Department of Pediatrics, Division of Pediatric Infectious Diseases, Faculty of Medicine, Universidad de Chile, Santiago, Chile; ^12^ Medical Research Council Unit The Gambia at London School of Hygiene and Tropical Medicine, Fajara, The Gambia; ^13^ All India Institute of Medical Sciences, New Delhi, India; ^14^ University of Jordan, Amman, Jordan; ^15^ Centre for Geographic Medicine Research-Coast, Kenya Medical Research Institute-Wellcome Trust Research Programme, Kilifi, Kenya; ^16^ School of Life Sciences, University of Warwick, Coventry, United Kingdom; ^17^ ISGlobal, Hospital Clínic - Universitat de Barcelona, Barcelona, Spain; ^18^ Centro de Investigação em Saúde de Manhiça (CISM), Maputo, Mozambique; ^19^ Institución Catalana de Investigación y Estudios Avanzados (ICREA), Barcelona, Spain; ^20^ Pediatrics Department, Hospital Sant Joan de Déu, Universitat de Barcelona, Barcelona, Spain; ^21^ Consorcio de Investigación Biomédica en Red de Epidemiología y Salud Pública (CIBERESP), Madrid, Spain; ^22^ Department of Paediatrics, Institute of Medicine, Tribhuvan University, Kathmandu, Nepal; ^23^ University of Bergen, Bergen, Norway; ^24^ Ahmadu Bello University/Teaching Hospital, Zaria, Nigeria; ^25^ Research Institute for Tropical Medicine, Muntinlupa, Philippines; ^26^ RITM-Tohoku Research Collaborating Center for Emerging Infections, Manila, Philippines; ^27^ South African Medical Research Council: Vaccines and Infectious Diseases Analytics Research Unit, University of the Witwatersrand, Johannesburg, South Africa; ^28^ Department of Science and Technology/National Research Foundation: Vaccine Preventable Diseases, Faculty of Health Sciences, University of the Witwatersrand, Johannesburg, South Africa; ^29^ Diagnostic and Research Virology Laboratory, Department of Microbiology, Faculty of Medicine, University of Peradeniya, Peradeniya, Sri Lanka; ^30^ Violicom Medical Limited, Aldermaston, United Kingdom; ^31^ Research Institute for Tropical Medicine, Muntinlupa, Philippines; ^32^ Department of Paediatrics and Child Health, Chris Hani Baragwanath Academic Hospital, Faculty of Health Sciences, University of the Witwatersrand, Johannesburg, South Africa; ^33^ Hospital Universitário da Universidade Federal do Maranhão, São Luís, Brazil; ^34^ Hospital Santa Casa de Ribeirão Preto, São Paulo, Brazil; ^35^ Centro Universitário Barão de Mauá, Ribeirão Preto, São Paulo, Brazil; ^36^ Hospital Militar Central, Bogotá, Colombia; ^37^ Neonatology Service, Hospital Clínic de Barcelona, Barcelona, Spain; ^38^ NYU Long Island School of Medicine, Mineola, NY, United States; ^39^ Hospital Santa Casa De São Paulo, São Paulo, Brazil; ^40^ Child Health and the SA-MRC Unit on Child and Adolescent Health, Department of Paediatrics, University of Cape Town (UCT), Cape Town, South Africa; ^41^ Ministry of Health and Population, Kathmandu, Nepal; ^42^ Center for Infectious Diseases Research, American University of Beirut, Beirut, Lebanon; ^43^ School of Medicine and Pharmacy, University Mohamed V, Rabat, Morocco; ^44^ National Public Health Laboratory, Ministry of Health & Population, Teku, Kathmandu, Nepal; ^45^ Universidade Federal do Amazonas, Manaus, Brazil; ^46^ Canadian Premature Babies Foundation, Toronto, ON, Canada; ^47^ National Hospital – Kandy, Kandy, Sri Lanka; ^48^ IRCCS-Sant'Orsola University Hospital, Bologna, Italy; ^49^ Hospital Clínico Universitario de Santiago, Universidade de Santiago, Santiago, Spain; ^50^ University of Calgary, Calgary, AB, Canada; ^51^ Department of Paediatrics and Child Health, University of Nairobi, Nairobi, Kenya; ^52^ Department of Pediatric Nursing, Maharajgunj Nursing Campus, Institute of Medicine, Tribhuwan University, Kathmandu, Nepal; ^53^ Pediatric Nurses Association of Nepal, Kathmandu, Nepal; ^54^ Nationwide Childrens Hospital, Columbus, OH, USA; ^55^ The Ohio State University, Columbus, OH, USA; ^56^ Hospital Materno-Infantil de Brasília, Brasília, Brazil; ^57^ Patan Academy of Health Sciences, Lalitpur, Nepal; ^58^ Department of Pediatrics, McMaster University, Hamilton, ON, Canada; ^59^ Surveillance Secretariat in Health, Ministry of Health, Brasilia, Brazil; ^60^ Ministry of Health Kenya, Nairobi, Kenya; ^61^ Bayero University/Aminu Kano Teaching Hospital, Kano, Nigeria; ^62^ University of Calabar/University of Calabar Teaching Hospital, Calabar, Nigeria; ^63^ Hospital de Niños Víctor J. Vilela de Rosario, Santa Fe, Argentina; ^64^ Hospital Nacional de Niños “Dr. Carlos Sáenz Herrera”, Caja Costarricense del Seguro Social (CCSS), San José, Costa Rica; ^65^ International Centre for Diarrhoeal Disease Research, Dhaka, Bangladesh; ^66^ University Hospital October 12, Madrid, Spain; ^67^ PSI Regional Technical Services Office, Nairobi, Kenya; ^68^ Metropolitan University of Santos, São Paulo, Brazil; ^69^ London Health Sciences Centre, London, ON, Canada; ^70^ Paropakar Maternity and Women's Hospital, Kathmandu, Nepal; ^71^ Hospital Sanatorio Trinidad, Buenos Aires, Argentina; ^72^ The Children's Hospital of Winnipeg, Winnipeg, MB, Canada; ^73^ McGill University, Montreal, QC, Canada; ^74^ General Sir John Kotelawala Defence University, Rathmalana, Sri Lanka; ^75^ University of Nigeria Teaching Hospital, Enugu, Nigeria; ^76^ Hospital Infantil Albert Sabin (HIAS), Fortaleza, Ceará, Brazil; ^77^ Usmanu Danfodiyo University Teaching Hospital, Sokoto, Nigeria; ^78^ Washington State University – Global Health Kenya, Nairobi, Kenya; ^79^ Orillia Soldiers’ Memorial Hospital, Orillia, Ontario, Canada; ^80^ Kenya Medical Research Institute- Center for Global Health Research, Centre for Geographic Medicine Research-Coast, Kilifi, Kenya; ^81^ Post Graduate Institute of Medical Education & Research, Chandigarh, India; ^82^ Ministry of Health Canada, Ottawa, ON, Canada; ^83^ Nepal Pediatric Society (NEPAS), Kanti Children's Hospital, Kathmandu, Nepal; ^84^ King George's Medical University, Uttar Pradesh, India

**Keywords:** RSV, developing countries, burden, diagnostics, management, prevention, decision research

## Abstract

**Introduction:**

The high burden of respiratory syncytial virus (RSV) infection in young children disproportionately occurs in low- and middle-income countries (LMICs). The PROUD (Preventing RespiratOry syncytial virUs in unDerdeveloped countries) Taskforce of 24 RSV worldwide experts assessed key needs for RSV prevention in LMICs, including vaccine and newer preventive measures.

**Methods:**

A global, survey-based study was undertaken in 2021. An online questionnaire was developed following three meetings of the Taskforce panellists wherein factors related to RSV infection, its prevention and management were identified using iterative questioning. Each factor was scored, by non-panellists interested in RSV, on a scale of zero (very-low-relevance) to 100 (very-high-relevance) within two scenarios: (1) Current and (2) Future expectations for RSV management.

**Results:**

Ninety questionnaires were completed: 70 by respondents (71.4% physicians; 27.1% researchers/scientists) from 16 LMICs and 20 from nine high-income (HI) countries (90.0% physicians; 5.0% researchers/scientists), as a reference group. Within LMICs, RSV awareness was perceived to be low, and management was not prioritised. Of the 100 factors scored, those related to improved diagnosis particularly access to affordable point-of-care diagnostics, disease burden data generation, clinical and general education, prompt access to new interventions, and engagement with policymakers/payers were identified of paramount importance. There was a strong need for clinical education and local data generation in the lowest economies, whereas upper-middle income countries were more closely aligned with HI countries in terms of current RSV service provision.

**Conclusion:**

Seven key actions for improving RSV prevention and management in LMICs are proposed.

## Introduction

Respiratory syncytial virus (RSV) lower respiratory tract infection (LRTI) causes over 3 million hospitalisations and over 100,000 deaths in children under 5 years every year (1, 2). Ninety-nine per cent of these fatalities occur in low- and middle-income countries (LMICs) where RSV has been reported to be the most frequent cause of mortality among infants beyond the neonatal period (1, 2). In light of this substantial burden, RSV LRTI prevention has been identified as a key priority by the World Health Organization (WHO) for the past 20 years (3–5). However, despite over 60 years of research, current preventive measures for RSV disease remain limited to good hygiene and the use of palivizumab, a monoclonal antibody used only for high-risk children, including premature infants (≤35 weeks' gestational age) and those with congenital heart disease and bronchopulmonary dysplasia (6). Additionally the use of palivizumab remains minimal in LMICs due to financial constraints and a lack of confidence among practitioners. This situation is expected to change in the next few years with several new preventive interventions under development, including long-acting monoclonal antibodies and maternal and infant vaccines (7).

Securing access to these interventions at a sustainable cost is often considered the prime concern relating to improving the management of RSV in LMICs (8). Indeed, WHO has stated that emerging vaccines and monoclonal antibodies should be made available to support optimal use in LMICs (9, 10). However, it should be recognised that there are other potential considerations and challenges in LMICs. These include limited access to healthcare, lack of awareness/understanding of the public health impact of RSV among healthcare professionals (HCPs) and policymakers, resource availability constraints, lack of reliable local/regional epidemiological and disease burden data to inform cost-effectiveness assessment and guide preventive efforts, and lack of access to point-of-care tests (8).

The PROUD (Preventing RespiratOry syncytial virUs in unDerdeveloped countries) Taskforce of 24 global RSV experts was established to help understand and propose solutions to these challenges. The Taskforce aims to lobby influential health providers, policymakers, public health organisations, and associations to work collaboratively to combat RSV in LMICs. As the first step to achieve this mission, the Taskforce undertook a detailed assessment of the key considerations and priorities for the prevention and management of RSV infection in LMICs with a global, online-based survey of HCPs and other key stakeholders (public health, policymakers, payers, *etc*) involved in RSV.

## Methods

### Study design

The study was conducted in 2021. The survey methodology encompassed two stages, first a qualitative stage to define the scope of the survey, followed by a quantitative stage to provide numerical data for objective analysis (11, 12).

#### Stage 1

The aim of stage one was to identify all the factors potentially related to the burden of RSV in LMICs, the challenges associated with this burden, and expectations for future management of RSV. This was accomplished by holding three online meetings (29th March; 1st April; 21st April 2021) with members of the PROUD Taskforce. At each meeting, five questions designed to stimulate thoughts about RSV, the burden it presents, and its current and future management in different, but complementary, situations were presented (Supplementary Material 1) and responses were gathered in sequence. Participants were given approximately 5 min to respond to each question. Individuals then took turns to read out their answers to the group to stimulate further suggestions from the other participants. The goal was to capture an exhaustive list of responses – data saturation – after going through all five questions. All responses were video recorded and documented. The responses from the meetings were compiled into a catalogue of distinct factors and used to construct a structured questionnaire (developed and approved by all Taskforce members), which constituted the second stage of the study (Supplementary Material 2).

#### Stage 2

The purpose of the questionnaire was to assess objectively the importance of each of the factors relative to LMICs when considering two defined scenarios: (1) The current situation regarding RSV management and, (2) Realistic future expectations for RSV management. The questionnaire was available in English on a secure online website (open from 17th August to 10th November 2021), a link to which was distributed *via* email by the Taskforce to HCPs, researchers, and others interested in RSV amongst their contacts in LMICs. Predicated on a previous study (11), a target of approximately 20 completed questionnaires was set for each of *Least Developed/Low-Income* (LD), *Low-Middle-Income* (LM), and *Upper-Middle-Income* (UM) countries, as defined by the Development Assistance Committee (DAC) List of Official Development Assistance Recipients (13). Representation from several countries withing each economic group was sought. A further approximately 20 completed questionnaires were sought from *High-Income* countries (HICs), to act as a reference group.

Within the questionnaire, each factor was scored on a continuous (parametric) end-anchored analogue scale from zero (very-low-relevance) to 100 (very-high-relevance) within both scenarios. The option to score any factor as “not relevant” was also provided. The order of the individual factors was randomised for scoring within each scenario to minimise unintended rationalisation of responses. Demographic details, including qualification/position, experience in RSV, and broad information on RSV testing and management in the respondent's country, were also captured on the questionnaire.

### Analysis of responses to stage 2 questionnaire

Questionnaire responses were analysed to address two key, interrelated questions:

1) What are the most important overall considerations for RSV management in LMICs?
2) What are the current vs. future priorities for RSV management in LMICs?

Question 1 was addressed by principal component analysis (PCA), with the two aforementioned scenarios as the dependent variables. PCA is a well-established technique for simplifying aggregate responses such that the component factors can be ranked from highest (most important) to lowest (least important) in terms of contribution to the variance across the questionnaire responses (14). Results were analysed for all LMICs combined and then by individual economic groups (LD, LM, UM, and HIC). The top quintile of factors was reported with related factors collated into areas/themes to aid interpretation, as agreed by the Taskforce. Differences between economies were explored further by calculating the relative contribution (loading) of each area/theme to explain the variance in the PCA for the top quintile of factors for each economic group. Question 2 was addressed using linear discriminant function analysis to maximise separation of the factors into those most closely associated with the current situation vs. future expectations, using the combined results for all LMICs.

Prior to analysis, missing data were imputed using the corresponding mean for that factor within each scenario. Factors scored as “not relevant” were assigned an analytically neutral value of 50. All analyses were carried out using SPSS for Windows 15.0 (IBM Corporation, New York, USA) and Excel 365 (Microsoft Corporation, Washington, USA).

### Role of the funding source

There was no funding source for this study.

### Ethics approval and informed consent

Ethics approval and patient consent was not required for this study. This study was a voluntary survey of clinicians and scientists regarding RSV infection, its prevention and management. No intervention was mandated, clinical practice was not affected and clinical data were not collected.

## Results

### Stage 1 – online meetings

Eighteen members of the Taskforce participated in the online meetings, 16 of whom represented LMICs (AG, NH, MS, JS, ShB, NKB, JN, PM, QB, AS, SB, MG, JDJC, SL, MN, and MD) and two (XCE and BP) from HICs. A catalogue of 100 individual factors potentially relevant to the current and future management of RSV in LMICs was generated (Supplementary Material 2).

### Stage 2 – questionnaire

Ninety questionnaires were completed, of which 70 were from LMICs and 20 from HICs (Table 1), after distribution to 198 people (45.5% response rate). Sixteen LMICs and nine HICs were represented in the survey (Supplementary Material 3). Overall, 34,600 numerical data points were generated for analysis, with 8% (7/90) of questionnaires returned incomplete (missing data: 1,400/36,000; 3.9%). The majority (50/70; 71%) of respondents from LMICs were medical doctors, with approximately half (37/70; 53%) having at least ten years' experience in the RSV field. Other respondents were primarily researchers and scientists, with LD and LM countries represented by a higher proportion of such professionals than UM and HICs. Knowledge/awareness of RSV within LMICs was perceived to be low by 39% (27/70) of respondents, and 23% (16/70) believed that RSV infection is afforded the necessary recognition as a priority public health issue. RSV testing for research/clinical management, was undertaken in LMICs by 43/70 (61%) of the respondents, primarily using polymerase chain reaction (PCR) tests. Palivizumab was reported to be locally available by nearly half (33/70; 47%) of respondents from LMICs, although an absence of support or public funding reduced equitable access for 48% (16/33) of these respondents. For the two lowest economic groups (LD and LM), knowledge/awareness of RSV, RSV prioritisation, RSV testing, and palivizumab publicly funded/support was notably lower than for UM countries, which were much closely aligned with HICs.

**Table 1:** Demographics and background information of respondents and the RSV situation in their countries.

Category	Response	LMICs			HICs^d^ (*n* = 20), %	All LMICs (*n* = 70), %
		LD^a^ (*n* = 18), %	LM^b^ (*n* = 31), %	UM^c^ (*n* = 21), %		
Occupation/qualification	Doctor	72.2	58.1	90.5	90.0	71.4
	Nurse	5.6	0.0	0.0	5.0	1.4
	Other^e^	22.2	41.9	9.5	5.0	27.1
Years of experience in RSV	None	5.6	0.0	0.0	0.0	1.4
	<1 year	5.6	6.5	0.0	0.0	4.3
	1-5 years	11.1	29.0	14.3	5.0	20.0
	6-10 years	27.8	19.4	19.0	0.0	21.4
	>10 years	50.0	45.1	66.7	95.0	52.9
Knowledge of RSV in your country	Low	44.4	54.8	9.5	5.0	38.6
	Moderate	50.0	41.9	81.0	65.0	55.7
	High	5.6	3.2	9.5	30.0	5.7
RSV a priority in your country	Yes	22.2	9.7	42.9	55.0	22.9
	No	61.1	83.8	57.1	30.0	70.0
	Do not know	16.7	6.5	0.0	15.0	7.1
RSV testing in your country	Yes	44.4	45.2	100.0	95.0	61.4
	No	50.0	48.3	0.0	5.0	34.3
	Do not know	5.6	6.5	0.0	0.0	4.3
If testing, type used	Immunofluorescence	5.6	12.9	57.1	40.0	24.3
	Antigen	0.0	9.7	33.3	70.0	14.3
	PCR	44.4	45.2	85.7	70.0	5.1
Use of palivizumab in your country	Yes	0.0	0.0	76.2	95.0	22.9
	Available but not supported/publicly funded	33.3	35.5	0.0	5.0	24.3
	No	27.8	41.9	23.8	0.0	32.9
	Do not know	38.9	22.6	0.0	0.0	20.0

HICs, high-income countries; LD, least developed/low-income countries; LM, lower-middle-income countries; LMICs, low- and middle-income countries; UM, upper-middle-income countries; PCR, polymerase chain reaction; RSV, respiratory syncytial virus.

^a^ Bangladesh, Gambia, Nepal.

^b^ India, Jordan, Kenya, Morocco, Nigeria, Philippines, Sri Lanka.

^c^ Argentina, Brazil, Colombia, Costa Rica, Lebanon, South Africa.

^d^ Australia, Canada, Chile, Italy, Netherlands, Norway, Spain, Sweden, United States.

^e^ Other occupation/qualification included: Scientist/Researcher/Clinical Officer (Assistant to Senior); Biomedic; Epidemiologist; Pharmacist; Public Health; Virologist.

#### Q1 – most important overall considerations for RSV management in LMICs

The single most important need identified for RSV management in LMICs was that for a simple, rapid, low-cost, point-of-care diagnostic test (Table 2), especially as testing access was considered limited, particularly in rural settings. The need for locally relevant data on the epidemiology and burden of RSV was also strongly represented, with two of the top five factors, and five of the top 20 factors being related to data generation on these topics, including establishing surveillance programmes, data on at-risk groups, and increasing understanding about the impact of co-infections. Affordability and access to future vaccines and prophylaxis and the need to improve service provision, including proactively establishing RSV vaccine delivery programmes and availability of oxygen and pulse oximeters, were also key themes. Other key factors identified related to clinical education, including the development of international, easy-to-follow, point-of-care guidelines, the need for a RSV vaccine, engagement with public health, policymakers and payers, and parent/public education. For the latter point, building on the lessons from coronavirus (COVID-19) to teach parents hygiene measures for avoiding RSV infection was scored highly.

**Table 2:** Top quintile of most important factors (*n* = 20) related to both current and future management of RSV in LMICs^a^.

Factor	Loading^b^
The need for a simple, rapid, low-cost point-of-care diagnostic technique	2.10
The need for research to generate more local epidemiological data on RSV	1.81
The need for an infant vaccine for RSV	1.61
The importance of a vaccine that is affordable and available to all	1.57
The need for a national surveillance programme for RSV	1.44
The need to establish an RSV national immunisation programme once a vaccine is available	1.36
The need for a cheaper widely applicable prophylactic treatment	1.35
Access to laboratory RSV diagnostic testing is limited especially in rural areas	1.31
The need to make oximeters available to all public hospitals and health facilities	1.24
The need for morbidity and mortality data as well as demographics of at-risk groups	1.22
Health burden of RSV not fully recognised by the public health bodies	1.13
The paucity of local data on the prevalence and burden of RSV in the community	1.08
The high cost of vaccines	1.07
The need for regular educational events, such as webinars, to update the important topic of paediatric and maternal vaccines for RSV	1.02
The need for internationally approved, easy-to-follow, point-of-care management guidelines for RSV	1.02
The need to improve the national supply chain for oxygen support especially in peripheral health centres	0.97
Build on the lessons of COVID-19 to teach parents hygiene measures for avoiding RSV infection	0.96
The need to understand more about co-infections with RSV since mortality may be increased	0.94
The need to keep policymakers on board with evolving RSV management/prevention strategies	0.93
Information on RSV needs to be widely available for the general population	0.89

COVID-19: coronavirus disease; RSV: respiratory syncytial virus.

^a^ Principal component analysis that contained 94.9% of the data variance.

^b^ The loading is the relative weighting of each factor within the principal component; individual loading comparisons are not extracted by PCA although the whole correlation matrix is highly significant (1-tailed) at the <0.001 level.

▪ Cost of and access to treatments, prophylaxis and vaccines ▪ Clinical education requirements (including the need for guidelines)

▪ Need for new preventive and treatment options ▪ Service provision and delivery

▪ Data gaps/areas for data generation ▪ Improving diagnosis

▪ Engaging with public health, policymakers and payers ▪ General education and awareness

**Figure 1:**
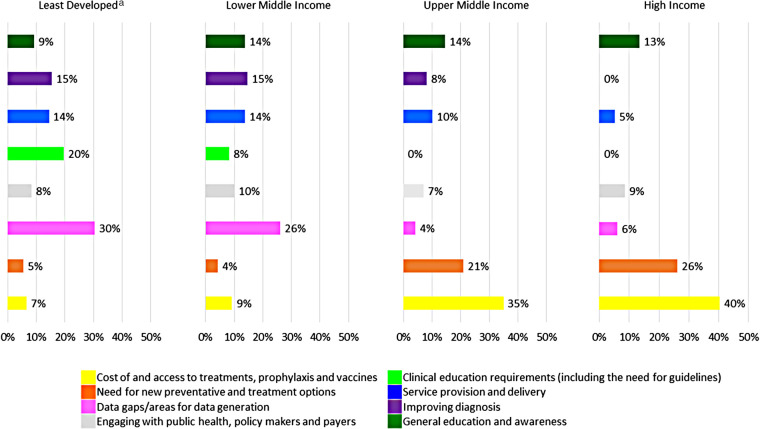
Most important areas related to the current and future management of respiratory syncytial virus categorised by economic group^b^. ^b^Bars represent the relative contribution (loading) of each area/theme to explaining the variance in the principal component analysis for the top quintile of factors for each economic group. ^a^Including low-income countries.

When analysed by economic status, a particular need was identified in LD and LM countries for local data generation and clinical education on RSV (Figure 1), including the need for training on RSV diagnosis and enabling distinction from bacterial infection (Supplementary Material 4). By contrast, the primary focus in UM and HICs was on the need for new preventive and treatment options for RSV, and their cost. Improving diagnosis and overall service provision was a marginally less pressing concern in UM than LD and LM countries. General education and awareness about RSV and engagement with key stakeholders appeared imperatives regardless of economic status.

#### Q2 – current and future priorities in RSV management in LMICs

All key areas identified as important for LMICs – improved diagnosis, data generation, clinical and general education, availability and access to new interventions, and engagement with policymakers/payers *etc* – were strongly associated with current RSV management (Table 3). Overall, more factors aligned with current (*n* = 19) than future RSV management (*n* = 7). Factors relevant to the current situation included the possibility of leveraging the experience of managing COVID-19 with policymakers to inform strategies for RSV prevention. However, it was also recognised that vaccines for other diseases might be prioritised over RSV vaccination. Future priorities centred around on-going education (6/7; 86%).

**Table 3:** Key factors distinguishing between current and future management of RSV in LMICs^a^.

Current RSV Management		Future Expectations for RSV Management	
Factor^b^	Loading^c^	Factor^b^	Loading^c^
*The fact that vaccines for other diseases may take priority over vaccination for RSV*	0.13	*The problems associated with RSV are particularly associated with lower socio-economic groups*	−0.06
The need to establish an RSV national immunisation programme once a vaccine is available	0.13	*Education by health professionals for mothers is a priority*	−0.06
*The need for education to raise awareness linking bronchiolitis to RSV infection*	0.12	*The lack of knowledge of evidence for the use of high flow nasal canulae in the management of RSV*	−0.06
*The need to target seasonal RSV infections*	0.12	*Recognising the long-term economic sequelae of RSV*	−0.04
The need for a cheaper widely applicable prophylactic treatment	0.12	*The need to cohort RSV patients in hospital wards*	−0.04
*The need for training on RSV diagnosis and enabling distinction from bacterial infection*	0.11	*Problem with translation of RSV education materials into local languages*	−0.03
The need for research to generate more local epidemiological data on RSV	0.11	*Need for recognition by stakeholders that respiratory tract infections in young children are mostly viral*	−0.022
*The need for RSV point-of-care testing to be routine practice*	0.11		
*Possibility of leveraging on the COVID-19 experience to persuade policymakers about strategies for RSV prevention*	0.11		
*Lack of general understanding of the healthcare costs during RSV hospitalisation*	0.11		
The need for a simple, rapid, low-cost point-of-care diagnostic technique	0.10		
The need for an infant vaccine for RSV	0.09		
*The need for an antiviral or other treatment which can be used for the management of RSV*	0.09		
*Need for leading experts to disseminate information on RSV*	0.09		
*The impact/burden on parents of an infant hospitalised with RSV is overlooked*	0.09		
*WHO guidelines tend to encourage the overuse of antibiotics based on an approach to the clinical diagnosis of all-cause pneumonia*	0.08		
*Worries about the acceptance of a RSV vaccine during pregnancy by conservative obstetric specialists*	0.08		
Information on RSV needs to be widely available for the general population	0.08		
Access to laboratory RSV diagnostic testing is limited especially in rural areas	0.08		

COVID-19: coronavirus disease; RSV: respiratory syncytial virus.

^a^ Discriminant function analysis that accounted for 100% of the variance.

^b^ Factors in italics were not identified in the top quintile of factors in the principal component analysis.

^c^ Loading score indicates the strength of the relationship between a factor and its scenario, with higher scores meaning closer association (for the purposes of the analysis the scenarios were assigned as positive and negative, but this has no bearing on interpretation of results).

▪ Cost of and access to treatments, prophylaxis and vaccines ▪ Clinical education requirements (including the need for guidelines)

▪ Need for new preventive and treatment options ▪ Service provision and delivery

▪ Data gaps/areas for data generation ▪ Improving diagnosis

▪ Engaging with public health, policymakers and payers ▪ General education and awareness

## Discussion

This study, led by the PROUD Taskforce, provides detailed insights into the significant considerations and priorities concerning the management and prevention of RSV in LMICs. Countries of the two lowest economic groups (LD and LM) were far more closely aligned with each other than with UM countries, which closely resembled HICs. Many of the same issues and needs were raised across all LMICs, and differences between economies predominantly related to focus or prioritisation. Predicated on the survey results, we propose seven key actions to effect change in LMICs (Table 4).

**Table 4:** Key actions for the prevention and management of RSV in LMICs.

1. Support the availability of simple, low-cost, point-of-care RSV diagnostic tests and develop and validate a scoring tool to aid diagnosis and severity assessment
2. Drive improvement in RSV management through ensuring the availability and appropriate use of oxygen therapy and oximeters
3. Support LMICs to generate local data on the epidemiology and burden of RSV
4. Increase knowledge about RSV among healthcare professionals and develop guidelines for RSV diagnosis and management
5. Build broader awareness of RSV among key non-clinical stakeholders
6. Support engagement with public health, policymakers, and payers
7. Prepare for rollout of vaccine/new single-dose monoclonal antibody

HCP, healthcare professional; LMIC, low- and middle-income countries; RSV, respiratory syncytial virus.

First, to support the availability of a simple, low-cost, point-of-care diagnostic test, which was identified as the most important need in LMICs (Table 2). This is a particular need in LD and LM countries, where 44.4% and 45.2%, respectively, reported that RSV testing was available, and this is likely concentrated in the larger urban hospitals and universities. Confirmatory diagnosis of RSV (and other viral infections) is important to help avoid the inappropriate use of antibiotics and the spread of multi-resistant bacteria in LMICs (15). However, increased provision of such diagnostic tests is unlikely to be a viable option in many LMICs due to cost and logistical constraints (8, 16). A potential solution, particularly in lower income areas, is the validation and adoption of a scoring tool to inform clinical diagnosis and assessment of RSV disease severity (8). Such a tool could combine demographic and clinical parameters (*e.g.,* age <6 months; infection during RSV season/during periods of increased detection; oxygen saturation <90%; tachypnoea; nasal flaring/grunting; apnoea; chest retractions; dehydration; poor feeding; cyanosis; lethargy; rales/rhonchi/wheezing) and prognostic biomarkers (17, 18). A meta-analysis or review of the currently available demographic and clinical profile data for children with RSV infection might serve as a baseline for development of a tool, and may guide local replication and data for validation. Additionally, the tool could be employed to target the use of RSV tests for confirmatory diagnosis and support local data generation.

Second, drive improvement in RSV management through availability and appropriate oxygen therapy and oximeters. LD and LM countries primarily focussed on improving the oxygen supply chain, while UM countries emphasised the need for more pulse oximeters (Supplementary Material 4). The importance of oxygen therapy and limited availability in LD countries is well-recognised and documented in a specific WHO report (19). We strongly advocate that LMICs be supported in their need for improved access to oxygen therapy, particularly considering the ongoing demands placed on respiratory support measures by COVID-19. Notably, the resolution of this issue depends on having the tools and expertise to make best use of the oxygen supply available (19, 20), and would be a core component covered in the clinical guidelines and education proposed below. Of note, oxygen administration *via* high-flow nasal cannulae can be very effective in severe cases and is a relatively simple technique. The emergence of COVID-19 has led to increased capacity building and logistical support, including oxygen supply for most countries, and extends an opportunity to leverage the existing enhanced infrastructure to better manage RSV.

Third, support LMICs to generate local data on the epidemiology and burden of RSV. Our findings indicate that the need for improved epidemiological and burden of illness data on RSV was driven primarily by the responses from LD and LM countries, although UM (and HICs) also expressed a desire for increased evidence (Figure 1). This aligns with a recent report indicating that only 54% (77/142) of LMICs have published data on the burden of RSV (8), and that routine surveillance for this virus may not be ongoing or sustainable. This shortfall perhaps relates to the challenges of obtaining a reliable diagnosis of RSV and capturing both hospital and community-based data, where most cases occur in the latter (2, 8, 21–23). Targeted efforts to address these data needs in LMICs are essential to support negotiations with policymakers and current and future payers (*e.g.,* Gavi, should a vaccine become available) and inform RSV immunisation and educational programmes for HCPs and the wider population. The WHO and Bill & Melinda Gates Foundation RSV surveillance programme has been initiated in 25 countries to support the introduction of RSV immunisation (24, 25), but more investment in this initiative is necessary. Strategies to gather and publish local data should be a focus for governments, healthcare providers, and non-governmental organisations (NGOs). The Child Health and Nutrition Research Initiative (CHNRI) method (26), a widely utilised framework to maximise return on research investment, was suggested to help prioritise the data generation.

Fourth, strongly support education, teaching and training for all relevant HCPs, including the development of simple, implementable RSV management guidelines. Given the comparatively low levels of knowledge and prioritisation of RSV in LD and LM countries vs. UM and HICs (Table 1), the survey highlighted a strong need for clinical education in countries in the two lowest economic groups (Figure 1). Two key factors were highlighted: the need for simple RSV guidelines and training on distinction from bacterial infection (Supplementary Material 4). There is widespread awareness of the WHO guideline for Integrated Management of Childhood Illness (IMCI) (27) in LMICs; however, the guideline focusses on early recognition and antibiotic treatment of childhood pneumonia, and RSV is not emphasised as a principal causal agent for LRTI. We recommend, therefore, that a specific RSV management guideline be developed, recognising resource and logistical restrictions prevalent in these countries that can be easily adapted and implemented at a local level. RSV experts should undertake development in partnership with key HCPs from the target countries to ensure their perspective is accurately captured while maximising the cultural and infrastructural relevance of the guidelines (28, 29). Utilisation of a Delphi methodology or other recognised consensus building approach is recommended to maximise robustness and applicability. Essential aspects to cover are information and updates on RSV vaccines and emerging monoclonal antibodies for RSV prevention (Table 2). Ideally, the proposed guideline will provide information on RSV infection as a significant cause of viral-related LRTI and support its addition to the new IMCI with appropriate management. A multichannel educational programme, available in a range of languages, involving webinars and modular learning programmes would permit flexibility in delivery.

Fifth, build broader awareness of RSV among non-clinical stakeholders. Increasing awareness of RSV and its associated burden amongst parents, payers, and public health stakeholders was a consistent need identified across all LMICs, including HICs (Figure 1). Overall, LD and LM countries recognised the need for basic RSV education, whereas the focus in UM countries and HICs was for more specific information on risk groups, and prevention strategies (Table 3 and Supplementary Material 4). This likely reflects differences in viewed priorities, with LD countries building their response to RSV from the ground up, whilst in more developed economies, the focus was on refining existing services. Lessons learnt from COVID-19, particularly the adoption of hygiene measures, were strongly advocated to prevent RSV infection (Table 2). Education of all key stakeholders was considered fundamental to driving change, improving management, and reducing the burden of RSV in LMICs.

Sixth, support engagement with public health, policymakers, and payers across all economic groups (Table 2 and Figure 1). The health burden of RSV was perceived to be not fully recognised by public health agencies in LMICs, particularly in LD and LM countries (Supplementary File 4), which indicates that RSV is not afforded the priority it richly deserves in these nations (Table 1). The actions detailed above all support engagement and lobbying for increased recognition and funding for RSV at a local level to improve current management, and pave the way for emerging RSV preventive modalities.

Lastly, development of a safe, effective RSV vaccine and new monoclonal antibodies and preparation for rollout. Whilst there was a strong desire for these preventive interventions, concerns about cost and how this could potentially limit access were raised by all economic groups (Table 2, Figure 1, and Supplementary Material 4). Proportionally, UM and HICs placed more emphasis on cost and access than LD and LM countries, reflecting that more work is needed in the latter in terms of building RSV awareness and service provision. WHO-led negotiations with the manufacturers will be critical to ensure equitable access in LMICs. Of the potential interventions, an infant vaccine received largest support from LMICs and, perhaps surprisingly, more support than maternal vaccination offering passive protection for at-risk infants. This may reflect the cost and logistical constraints apparent with palivizumab prophylaxis and the success of other global infant vaccines (*e.g.,* polio). Interestingly, a recently published retrospective analysis of a randomised controlled trial reported that over the first 3 months of life, maternal vaccination prevented 5.1 antimicrobial courses *per* 100 infants in LMICs, representing 10.9% of all antimicrobial prescribing (30). Hence, the availability of a maternal vaccine may have the potential to reduce the (over)reliance on antibiotic treatment of childhood pneumonia in these countries. The need to establish national RSV prevention programmes once a vaccine and other therapeutic interventions are available was strongly expressed; partnership with Gavi, the Vaccine Alliance, will be essential. The COVID-19 pandemic potentially offers an excellent opportunity to enhance immunisation programmes for respiratory viruses, including setting-up a similar scheme to COVAX for RSV (31).

Several limitations of the survey should be recognised. Respondents were first chosen by the Taskforce members based on known interest in RSV, and then on their willingness to complete the survey, which poses a potential selection bias. The respondents were also primarily physicians and researchers from leading national centres, reflecting the membership of the Taskforce and their contacts. These factors were not considered significant limitations, as the goal was to gain a detailed understanding of the priorities and needs within LMICs, which the respondents were certainly able to provide. The questionnaire being available only in English is another potential limitation, although a single version of the questionnaire was preferable to multiple translations. Finally, completer bias may be relevant because of the lengthy questionnaire (background information plus scoring 100 factors across two scenarios). However, missing data (3.9%) was minimal, so replacement with mean values is unlikely to have significantly influenced the results. Furthermore, the use of three meetings to generate a list of items, scoring them for relevance, and randomising the factors within the two scenarios helped mitigate any tendency to rationalise responses, which may introduce bias in surveys (32). Moreover, the methodology employed for the survey started with no predetermined questions but generated an exhaustive list of factors which is a major strength. It should also be recognised that the findings are in-line with other similar studies, supporting their veracity (8, 9, 20).

Our study has provided a novel, LMIC-led perspective on the major considerations and priorities to improve the management and reduce the substantial burden of RSV infection in LMICs, and its impact on children. Seven key actions have been proposed, all of which are eminently achievable. We call upon the support of WHO, NGOs, and other key stakeholders to make them a reality.

## Data Availability

The raw data supporting the conclusions of this article will be made available by the authors, without undue reservation.

## PROUD taskforce

XCE, BAP, LJB, AG, NH, MS, RTS, JPTT, JS, SB, NK-B, DJN, PKM, QB, AS, SB, MG, JDJ, SPL, MCN, MD, JRF, BSR, EAFS.
