# Supplementary material for: Identifying the research, advocacy, policy and implementation needs for the prevention and management of respiratory syncytial virus lower respiratory tract infection in low- and middle-income countries
Source: Front Pediatr. 2022 Nov 9;10:1033125. doi: 10.3389/fped.2022.1033125 (PMC9682277; doi:10.3389/fped.2022.1033125)
Supplement: Supplementary file 1 [file Datasheet1.pdf]

Supplementary Materials

Supplementary File 1: Stage One Meeting Questions…………………………………………2

Supplementary File 2: Stage Two Questionnaire……………………………………….……..3

Supplementary File 3: WHO regions and countries represented by respondents in each economic group…………………………………………………………………………....33

Supplementary File 4: Top quintile of most important factors (n=20) related to the current and future management of RSV split by economic groupings………………………………34

Supplementary File 1: Stage One Meeting Questions

Question 1

- Write down anything that comes to mind when thinking about the particular difficulties in managing and controlling RSV in your country
  - Remember to include things that you think may be considered as personal, unusual, trivial or unique to your circumstances

Question 2

- Write down anything that comes to mind when thinking about the priorities in managing and controlling RSV in your country
  - Remember to include things that you think may be considered as personal, unusual, trivial or unique to your circumstances

Question 3

- Write down anything that comes to mind when thinking about the most promising ideas for managing and controlling RSV in your country
  - Remember to include things that you think may be considered as personal, unusual, trivial or unique to your circumstances

Question 4

- Write down anything that comes to mind when thinking about the opportunities to improve the management and control of RSV in your country
  - Remember to include things that you think may be considered as personal, unusual, trivial or unique to your circumstances

Question 5

- Write down anything that comes to mind when thinking about the realistic expectations for future management and control of RSV in your country
  - Remember to include things that you think may be considered as personal, unusual, trivial or unique to your circumstances

Supplementary File 2: Stage Two Questionnaire

Preventing **R**espirat**O**ry syncytial vir**U**s in un**D**erdeveloped countries

Understanding the Burden and Management of RSV in LMIC Countries

Decision Research Questionnaire

V1.0

Background Information

Introduction

We have an ambitious aim that we are very passionate about which is to create a global effort and program to abolish the terrible burden of respiratory syncytial virus (RSV) disease in babies and infants in low- and lower-middle income countries (LMICs). We take inspiration from what has been done with ending polio through the Rotary campaign and want to achieve a similar world-wide goal with RSV. We feel that this is the perfect time to start such a campaign with several vaccines, antibodies, and treatments for RSV on the near horizon.

To accomplish this, we have formed the PROUD (**P**reventing **R**espirat**O**ry syncytial vir**U**s in un**D**erdeveloped countries) Task Force – a powerful opinion group of RSV experts from around the world.

We are taking a stepwise approach towards the achievement of our objectives. The purpose of this questionnaire is to better understand and quantify the impact of RSV and future expectations in RSV management in LMICs. The results of this research will be published in a peer-reviewed journal and <u>we would welcome you to be an author on the paper</u>.

Our goal is for maximum impact to guide and try to convince Health Providers, Associations with global influence, well-reputed Institutions and RSV networks of the importance of contemplating this problem globally and that the current reality shows us where the true medical and economic burden is – in LMICs!

Thank you for your support

Xavier Carbonell-Estrany (Spain) | Bosco Paes (Canada) | Louis Bont (Netherlands) | Eric Simoes (USA)

Angela Gentile (Argentina) | Nusrat Homaira (Bangladesh) | Marcelo Scotta (Brazil) | Renato Stein (Brazil) | Jarju Sheikh (Gambia) | Shobha Broor (India) | Najwa Khuri-Bulos (Jordan) | James Nokes (Kenya) | Patrick Munywoki (Kenya) | Quique Bassat (Mozambique) | Arun Sharma (Nepal) | Sudha Basnet (Nepal) | John Fullarton (New Zealand) | Maria Garba (Nigeria) | Socorro Lupisan (Philippines) | Joanne De Jesus-Cornejo (Philippines) | Marta Nunes (South Africa) | Maduja Divaratne (Sri Lanka) | Barry Rodgers-Gray (UK)

**1***	Name: ______________________________ Institution: ______________________________ E-mail______________________

Background Information

**2**	Country:[†] ____
**3**	What is you qualification/position?<br>☐ Doctor ☐ Nurse ☐ Other If Other, please describe____
**4**	How many years of experience do you have in the field of RSV?<br>☐ <1 year ☐ 1-5 years ☐ 6-10 years ☐ >10 years ☐ none
**5**	How highly would you rate the degree of knowledge by general practitioners, paediatricians and institutions of the RSV burden in your country?<br>☐ Low ☐ Moderate ☐ High
**6**	Is RSV considered a priority in your country by government and public health bodies?<br>☐ Yes ☐ No ☐ Don't know
**7**	Is RSV diagnostic testing available in your country?<br>☐ Yes ☐ No ☐ Don't know<br><br>If Yes, what tests are used:<br>☐ Direct immunofluorescence assays ☐ Automated rapid antigen detection tests ☐ Reverse-transcription polymerase chain reaction<br>If others, please describe____
**8**	Is palivizumab (Synagis) used in your country to prevent RSV?<br>☐ Yes ☐ No (available but use not supported/publicly funded) ☐ Not available ☐ Don't know<br><br>If Yes, for which groups:<br>☐ Bronchopulmonary dysplasia (BPD)/chronic lung disease (CLD) ☐ Congenital heart disease (CHD) ☐ Other serious medical disorders<br>☐ All premature infants ≤35 weeks' gestational age (wGA) or only ☐ ≤29 wGA ☐ 30-32 wGA ☐ 33-35 wGA
**9**	Who are the key stakeholder/decision makers in your country regarding public health policy and prevention of RSV?<br>____

**Please complete to be added as an author on the paper based on the research; [†]This should be the country you are representing with your answers, not the country you are currently based, if you have relocated*

About Decision Research

Background Information

Decision Research is a research tool with a proven methodology that can provide powerful insight into decision making and understanding the impact of a disease and its treatment. This questionnaire was developed following three 'Cataloguing' sessions with the PROUD Taskforce wherein a list of all possible items related to the current and future prevention and treatment of RSV infection in children was created (the 'Catalogue'). At each Cataloguing session, members of the Task Force were asked a series of overlapping questions about RSV in children, the responses to which were then discussed and expanded upon. The outputs from the three sessions were then amalgamated and condensed to form the final Catalogue. The questionnaire is designed to measure, in an objective manner, the relevance in your country of each of the items in the Catalogue. This will provide us with a rich dataset for analysis, enabling an in-depth understanding of the burden and management of RSV in LMICs.

For more information on the Decision Research process, please see: Carbonell-Estrany X et al. Acta Paediatr 2018;107:854-860.

Instructions

There are two sections or parts to the remainder of the questionnaire:

- Section 1 – the current situation regarding RSV management in your country
- Section 2 – realistic future expectations for RSV management in your country

For each section, please answer each question by making a vertical mark on the line (visual analogue scale) next to the question to indicate how relevant you think that particular issue is in your country (or country you are representing if relocated).

For example:

	Relevance	
Recognising the long-term economic sequelae of RSV	very low ________________________I________________ very high	☐ Not relevant
The need for longer term follow-up for RSV patients	very low ___________________I______________________ very high	☐ Not relevant

Please note that:

- In each section, the Catalogue items are repeated – this will enable multivariate analysis on the relative importance of and interrelationships between the items within each context (current situation, realistic expectations, and ideal hopes)
- The Catalogue items are randomised – this reduces any (unintentional) rationalisation of answers and reduces visual analogue scale fatigue
- The visual analogue scale is deliberately not numbered – this avoids the tendency for number preference
- There is the option of marking an item as 'Not relevant' if not applicable to the situation in your country
- This questionnaire should take 30-45 minutes to complete

Please do not spend too much time thinking about each item – your immediate reaction is what we are after!

SECTION 1: Current situation regarding RSV management

When you think about:

the current situation regarding RSV management in your country

How relevant are the following items to you?

Item	Relevance	
Education on RSV needs to be improved amongst paediatricians	very low________________________very high	☐ Not relevant
The need for regular educational events, such as webinars, to update the important topic of paediatric and maternal vaccines for RSV	very low________________________very high	☐ Not relevant
Poor appreciation of the efficacy of prophylactic treatments	very low________________________very high	☐ Not relevant
The need for internationally approved, easy to follow, point of care management guidelines for RSV	very low________________________very high	☐ Not relevant
The lack of approved strategies for the treatment of high-risk infants	very low________________________very high	☐ Not relevant
Lack of ICU (intensive care) beds	very low________________________very high	☐ Not relevant
Recognising the long-term economic sequelae of RSV	very low________________________very high	☐ Not relevant
Education by health professionals for mothers is a priority	very low________________________very high	☐ Not relevant
RSV is a viral infection without a specific medication/treatment	very low________________________very high	
Education on RSV needs to be improved amongst urban family physicians and rural doctors	very low________________________very high	☐ Not relevant
Understanding that RSV can be a problem in children with particular co-morbidities	very low________________________very high	☐ Not relevant
The need to make oximeters available to all public hospitals and health facilities	very low________________________very high	☐ Not relevant
WHO guidelines tend to encourage the overuse of antibiotics based on an approach to the clinical diagnosis of all-cause pneumonia	very low________________________very high	☐ Not relevant

SECTION 1: Current situation regarding RSV management

The need to cohort RSV patients in hospital wards	very low_______________________very high	☐ Not relevant
The importance of breast-feeding as a defence against RSV	very low_______________________very high	☐ Not relevant
The problems associated with RSV are particularly associated with lower socio-economic groups	very low_______________________very high	☐ Not relevant
Inability to rule out bacterial co-infection with RSV which requires antibiotics	very low_______________________very high	☐ Not relevant
Need for recognition by stakeholders that respiratory tract infections in young children are mostly viral	very low_______________________very high	☐ Not relevant
The lack of knowledge of evidence for the use of high flow nasal canulae in the management of RSV	very low_______________________very high	☐ Not relevant
Worry about declining breast-feeding rates	very low_______________________very high	☐ Not relevant
The importance of timing for any new maternal vaccine for RSV during pregnancy	very low_______________________very high	☐ Not relevant
Wards are crowded during the peak RSV season	very low_______________________very high	☐ Not relevant
Difficulty of achieving a common consensus on optimal RSV management between hospital specialists (lack of guidelines)	very low_______________________very high	☐ Not relevant
The potential high cost of new palivizumab biosimilar prophylaxis products	very low_______________________very high	☐ Not relevant
The importance of a vaccine that is affordable and available to all	very low_______________________very high	☐ Not relevant
The need for a national surveillance programme for RSV	very low_______________________very high	☐ Not relevant
The availability of an intra-nasal vaccine for RSV	very low_______________________very high	☐ Not relevant
Build on the lessons of COVID to teach parents hygiene measures for avoiding RSV infection	very low_______________________very high	☐ Not relevant

SECTION 1: Current situation regarding RSV management

The fact that not enough is known about the aetiology and pathogenesis of RSV	very low________________________very high	☐ Not relevant
The need for morbidity and mortality data as well as demographics of at-risk groups	very low________________________very high	☐ Not relevant
The need to understand more about co-infections with RSV since mortality may be increased	very low________________________very high	☐ Not relevant
The problem of misdiagnosis of RSV as bacterial infection	very low________________________very high	☐ Not relevant
The high cost of prophylactic monoclonal antibody treatment, including even biosimilars	very low________________________very high	☐ Not relevant
The need to keep policy makers on board with evolving RSV management/prevention strategies	very low________________________very high	☐ Not relevant
Logistic problems establishing special prevention clinics	very low________________________very high	☐ Not relevant
Access to laboratory RSV diagnostic testing is limited especially in rural areas	very low________________________very high	☐ Not relevant
Parents may not be able to gain access to hospital care because of employment or transport issues	very low________________________very high	☐ Not relevant
The need for strategies to boost the infant's immune system such as diet, healthy lifestyle	very low________________________very high	☐ Not relevant
The need for training on RSV diagnosis and enabling distinction from bacterial infection	very low________________________very high	☐ Not relevant
The difficulty associated with the logistics of administering prophylaxis to all children	very low________________________very high	☐ Not relevant
Involving local medicine vendors into the education rollout	very low________________________very high	☐ Not relevant
The need for RSV prevention in all children not just those at high risk	very low________________________very high	☐ Not relevant
Average age for RSV infection is in the very young	very low________________________very high	☐ Not relevant

SECTION 1: Current situation regarding RSV management

Recognising the long-term clinical sequelae of RSV	very low____________________very high	☐ Not relevant
The need for a cheaper widely applicable prophylactic treatment	very low____________________very high	☐ Not relevant
Possibility of leveraging on the COVID experience to persuade policy makers about strategies for RSV prevention	very low____________________very high	☐ Not relevant
Integrate information/education on RSV amongst healthcare providers and general population for countrywide acceptance	very low____________________very high	☐ Not relevant
The peak RSV season varies across the country	very low____________________very high	☐ Not relevant
The paucity of local data on the prevalence and burden of RSV in the community	very low____________________very high	☐ Not relevant
Health burden of RSV not fully recognised by the public health bodies	very low____________________very high	☐ Not relevant
Lack of understanding of the risk factors and preventative measures for RSV	very low____________________very high	☐ Not relevant
The need to recognise house overcrowding as a major risk for RSV	very low____________________very high	☐ Not relevant
The need to recognise pollution as a major risk for RSV	very low____________________very high	☐ Not relevant
The need to emphasise the importance of local supportive care for RSV	very low____________________very high	☐ Not relevant
The need for longer term follow-up for RSV patients	very low____________________very high	☐ Not relevant
The need to improve the national supply chain for oxygen support especially in peripheral health centres	very low____________________very high	☐ Not relevant
The need to recognise smoking as a major risk factor for RSV	very low____________________very high	☐ Not relevant
The issue of high patient to HCP (healthcare professional) ratio	very low____________________very high	☐ Not relevant
The impact/burden on parents of an infant hospitalised with RSV is overlooked	very low____________________very high	☐ Not relevant
Dissemination of information on the local burden of RSV	very low____________________very high	☐ Not relevant

SECTION 1: Current situation regarding RSV management

The fact that vaccines for other diseases may take priority over vaccination for RSV	very low____________________very high	☐ Not relevant
The need to establish an RSV national immunisation programme once a vaccine is available	very low____________________very high	☐ Not relevant
Information on RSV needs to be widely available for the general population	very low____________________very high	☐ Not relevant
The need to ensure that paediatricians understand the importance of and can properly manage oxygen therapy	very low____________________very high	☐ Not relevant
The need for a single dose long-acting prophylactic monoclonal treatment for RSV	very low____________________very high	☐ Not relevant
The need for RSV point of care testing to be routine practice	very low____________________very high	☐ Not relevant
The need for public and especially parental education on RSV and its symptoms	very low____________________very high	☐ Not relevant
The worry that new strategies for RSV prophylaxis and treatment will be unaffordable	very low____________________very high	☐ Not relevant
RSV not a priority for the public health bodies	very low____________________very high	☐ Not relevant
The importance of the duration of vaccine protection	very low____________________very high	☐ Not relevant
The lack of a health insurance system or national health service prevents local progress in managing RSV	very low____________________very high	☐ Not relevant
The need for a good prognosis/risk stratification tool to target RSV interventions	very low____________________very high	☐ Not relevant
The need to target seasonal RSV infections	very low____________________very high	☐ Not relevant
The importance of international vaccine programmes such as GAVI	very low____________________very high	☐ Not relevant

SECTION 1: Current situation regarding RSV management

The high cost of vaccines	very low____________________very high	☐ Not relevant
Children may be given bronchodilators even if RSV is recognised as a viral infection	very low____________________very high	☐ Not relevant
Lack of awareness about the morbidity and mortality associated with RSV	very low____________________very high	☐ Not relevant
Need for earlier referrals from primary to tertiary care centres	very low____________________very high	☐ Not relevant
The need for an infant vaccine for RSV	very low____________________very high	☐ Not relevant
Lack of general understanding of the healthcare costs during RSV hospitalisation	very low____________________very high	☐ Not relevant
Poor appreciation of the safety of prophylactic treatments	very low____________________very high	☐ Not relevant
Need for leading experts to disseminate information on RSV	very low____________________very high	☐ Not relevant
Problem with translation of RSV education materials into local languages	very low____________________very high	☐ Not relevant
WHO guidelines encourage use of antibiotics for respiratory infections based on an approach to the clinical diagnosis of all-cause pneumonia	very low____________________very high	☐ Not relevant
Training and education of relevant stakeholders is required to motivate and set RSV as a priority	very low____________________very high	☐ Not relevant
Poor appreciation of the potential efficacy of combined interventions such as maternal vaccine plus monoclonal prophylaxis	very low____________________very high	☐ Not relevant
Unequal access to resources for RSV countrywide especially between urban and rural settings	very low____________________very high	☐ Not relevant
Worries about the acceptance of a pregnancy RSV vaccine by conservative obstetric specialists	very low____________________very high	☐ Not relevant
Involve professional associations in creating awareness of and providing advocacy for RSV	very low____________________very high	☐ Not relevant

SECTION 1: Current situation regarding RSV management

Worries about the acceptability of vaccine for pregnant mothers especially in some communities	very low____very high	☐ Not relevant
The availability of a maternal vaccine for RSV	very low____very high	☐ Not relevant
Community acquired RSV infections not getting referred to hospital in a timely manner	very low____very high	☐ Not relevant
The need for an antiviral or other treatment which can be used for the management of RSV	very low____very high	☐ Not relevant
The lack of emphasis by healthcare information services on the prevention of RSV	very low____very high	☐ Not relevant
Tailored approach for RSV is required for different populations	very low____very high	☐ Not relevant
The need for expectant mothers to be taught alarm signs for RSV in antenatal clinics	very low____very high	☐ Not relevant
The need for research to generate more local epidemiological data on RSV	very low____very high	☐ Not relevant
The need for a simple, rapid, low-cost point of care diagnostic technique	very low____very high	☐ Not relevant
The need for education to raise awareness linking bronchiolitis to RSV infection	very low____very high	☐ Not relevant
During the RSV season, children hospitalised with RSV prevent hospitalisation of non-RSV patients	very low____very high	☐ Not relevant

End of SECTION 1

SECTION 2: Realistic future expectations for RSV management

When you think about:

realistic future expectations for RSV management in your country

How relevant are the following items to you?

	Relevance	
Education on RSV needs to be improved amongst paediatricians	very low________________________very high	☐ Not relevant
The need for regular educational events, such as webinars, to update the important topic of paediatric and maternal vaccines for RSV	very low________________________very high	☐ Not relevant
Poor appreciation of the efficacy of prophylactic treatments	very low________________________very high	☐ Not relevant
The need for internationally approved, easy to follow, point of care management guidelines for RSV	very low________________________very high	☐ Not relevant
The lack of approved strategies for the treatment of high-risk infants	very low________________________very high	☐ Not relevant
Lack of ICU (intensive care) beds	very low________________________very high	☐ Not relevant
Recognising the long-term economic sequelae of RSV	very low________________________very high	☐ Not relevant
Education by health professionals for mothers is a priority	very low________________________very high	☐ Not relevant
RSV is a viral infection without a specific medication/treatment	very low________________________very high	
Education on RSV needs to be improved amongst urban family physicians and rural doctors	very low________________________very high	☐ Not relevant
Understanding that RSV can be a problem in children with particular co-morbidities	very low________________________very high	☐ Not relevant
The need to make oximeters available to all public hospitals and health facilities	very low________________________very high	☐ Not relevant
WHO guidelines tend to encourage the overuse of antibiotics based on an approach to the clinical diagnosis of all-cause pneumonia	very low________________________very high	☐ Not relevant

SECTION 2: Realistic future expectations for RSV management

The need to cohort RSV patients in hospital wards	very low__________________very high	☐ Not relevant
The importance of breast-feeding as a defence against RSV	very low__________________very high	☐ Not relevant
The problems associated with RSV are particularly associated with lower socio-economic groups	very low__________________very high	☐ Not relevant
Inability to rule out bacterial co-infection with RSV which requires antibiotics	very low__________________very high	☐ Not relevant
Need for recognition by stakeholders that respiratory tract infections in young children are mostly viral	very low__________________very high	☐ Not relevant
The lack of knowledge of evidence for the use of high flow nasal canulae in the management of RSV	very low__________________very high	☐ Not relevant
Worry about declining breast-feeding rates	very low__________________very high	☐ Not relevant
The importance of timing for any new maternal vaccine for RSV during pregnancy	very low__________________very high	☐ Not relevant
Wards are crowded during the peak RSV season	very low__________________very high	☐ Not relevant
Difficulty of achieving a common consensus on optimal RSV management between hospital specialists (lack of guidelines)	very low__________________very high	☐ Not relevant
The potential high cost of new palivizumab biosimilar prophylaxis products	very low__________________very high	☐ Not relevant
The importance of a vaccine that is affordable and available to all	very low__________________very high	☐ Not relevant
The need for a national surveillance programme for RSV	very low__________________very high	☐ Not relevant
The availability of an intra-nasal vaccine for RSV	very low__________________very high	☐ Not relevant
Build on the lessons of COVID to teach parents hygiene measures for avoiding RSV infection	very low__________________very high	☐ Not relevant

SECTION 2: Realistic future expectations for RSV management

The fact that not enough is known about the aetiology and pathogenesis of RSV	very low________________________________very high	☐ Not relevant
The need for morbidity and mortality data as well as demographics of at-risk groups	very low________________________________very high	☐ Not relevant
The need to understand more about co-infections with RSV since mortality may be increased	very low________________________________very high	☐ Not relevant
The problem of misdiagnosis of RSV as bacterial infection	very low________________________________very high	☐ Not relevant
The high cost of prophylactic monoclonal antibody treatment, including even biosimilars	very low________________________________very high	☐ Not relevant
The need to keep policy makers on board with evolving RSV management/prevention strategies	very low________________________________very high	☐ Not relevant
Logistic problems establishing special prevention clinics	very low________________________________very high	☐ Not relevant
Access to laboratory RSV diagnostic testing is limited especially in rural areas	very low________________________________very high	☐ Not relevant
Parents may not be able to gain access to hospital care because of employment or transport issues	very low________________________________very high	☐ Not relevant
The need for strategies to boost the infant's immune system such as diet, healthy lifestyle	very low________________________________very high	☐ Not relevant
The need for training on RSV diagnosis and enabling distinction from bacterial infection	very low________________________________very high	☐ Not relevant
The difficulty associated with the logistics of administering prophylaxis to all children	very low________________________________very high	☐ Not relevant
Involving local medicine vendors into the education rollout	very low________________________________very high	☐ Not relevant
The need for RSV prevention in all children not just those at high risk	very low________________________________very high	☐ Not relevant

SECTION 2: Realistic future expectations for RSV management

Average age for RSV infection is in the very young	very low____	very high	☐ Not relevant
Recognising the long-term clinical sequelae of RSV	very low____	very high	☐ Not relevant
The need for a cheaper widely applicable prophylactic treatment	very low____	very high	☐ Not relevant
Possibility of leveraging on the COVID experience to persuade policy makers about strategies for RSV prevention	very low____	very high	☐ Not relevant
Integrate information/education on RSV amongst healthcare providers and general population for countrywide acceptance	very low____	very high	☐ Not relevant
The peak RSV season varies across the country	very low____	very high	☐ Not relevant
The paucity of local data on the prevalence and burden of RSV in the community	very low____	very high	☐ Not relevant
Health burden of RSV not fully recognised by the public health bodies	very low____	very high	☐ Not relevant
Lack of understanding of the risk factors and preventative measures for RSV	very low____	very high	☐ Not relevant
The need to recognise house overcrowding as a major risk for RSV	very low____	very high	☐ Not relevant
The need to recognise pollution as a major risk for RSV	very low____	very high	☐ Not relevant
The need to emphasise the importance of local supportive care for RSV	very low____	very high	☐ Not relevant
The need for longer term follow-up for RSV patients	very low____	very high	☐ Not relevant
The need to improve the national supply chain for oxygen support especially in peripheral health centres	very low____	very high	☐ Not relevant
The need to recognise smoking as a major risk factor for RSV	very low____	very high	☐ Not relevant
The issue of high patient to HCP (healthcare professional) ratio	very low____	very high	☐ Not relevant
The impact/burden on parents of an infant hospitalised with RSV is overlooked	very low____	very high	☐ Not relevant

SECTION 2: Realistic future expectations for RSV management

Dissemination of information on the local burden of RSV	very low________________________very high	☐ Not relevant
The fact that vaccines for other diseases may take priority over vaccination for RSV	very low________________________very high	☐ Not relevant
The need to establish an RSV national immunisation programme once a vaccine is available	very low________________________very high	☐ Not relevant
Information on RSV needs to be widely available for the general population	very low________________________very high	☐ Not relevant
The need to ensure that paediatricians understand the importance of and can properly manage oxygen therapy	very low________________________very high	☐ Not relevant
The need for a single dose long-acting prophylactic monoclonal treatment for RSV	very low________________________very high	☐ Not relevant
The need for RSV point of care testing to be routine practice	very low________________________very high	☐ Not relevant
The need for public and especially parental education on RSV and its symptoms	very low________________________very high	☐ Not relevant
The worry that new strategies for RSV prophylaxis and treatment will be unaffordable	very low________________________very high	☐ Not relevant
RSV not a priority for the public health bodies	very low________________________very high	☐ Not relevant
The importance of the duration of vaccine protection	very low________________________very high	☐ Not relevant
The lack of a health insurance system or national health service prevents local progress in managing RSV	very low________________________very high	☐ Not relevant
The need for a good prognosis/risk stratification tool to target RSV interventions	very low________________________very high	☐ Not relevant
The need to target seasonal RSV infections	very low________________________very high	☐ Not relevant
The importance of international vaccine programmes such as GAVI	very low________________________very high	☐ Not relevant

SECTION 2: Realistic future expectations for RSV management

The high cost of vaccines	very low____very high	☐ Not relevant
Children may be given bronchodilators even if RSV is recognised as a viral infection	very low____very high	☐ Not relevant
Lack of awareness about the morbidity and mortality associated with RSV	very low____very high	☐ Not relevant
Need for earlier referrals from primary to tertiary care centres	very low____very high	☐ Not relevant
The need for an infant vaccine for RSV	very low____very high	☐ Not relevant
Lack of general understanding of the healthcare costs during RSV hospitalisation	very low____very high	☐ Not relevant
Poor appreciation of the safety of prophylactic treatments	very low____very high	☐ Not relevant
Need for leading experts to disseminate information on RSV	very low____very high	☐ Not relevant
Problem with translation of RSV education materials into local languages	very low____very high	☐ Not relevant
WHO guidelines encourage use of antibiotics for respiratory infections based on an approach to the clinical diagnosis of all-cause pneumonia	very low____very high	☐ Not relevant
Training and education of relevant stakeholders is required to motivate and set RSV as a priority	very low____very high	☐ Not relevant
Poor appreciation of the potential efficacy of combined interventions such as maternal vaccine plus monoclonal prophylaxis	very low____very high	☐ Not relevant
Unequal access to resources for RSV countrywide especially between urban and rural settings	very low____very high	☐ Not relevant
Worries about the acceptance of a pregnancy RSV vaccine by conservative obstetric specialists	very low____very high	☐ Not relevant

SECTION 2: Realistic future expectations for RSV management

Involve professional associations in creating awareness of and providing advocacy for RSV	very low__________________________very high	☐ Not relevant
Worries about the acceptability of vaccine for pregnant mothers especially in some communities	very low__________________________very high	☐ Not relevant
The availability of a maternal vaccine for RSV	very low__________________________very high	☐ Not relevant
Community acquired RSV infections not getting referred to hospital in a timely manner	very low__________________________very high	☐ Not relevant
The need for an antiviral or other treatment which can be used for the management of RSV	very low__________________________very high	☐ Not relevant
The lack of emphasis by healthcare information services on the prevention of RSV	very low__________________________very high	☐ Not relevant
Tailored approach for RSV is required for different populations	very low__________________________very high	☐ Not relevant
The need for expectant mothers to be taught alarm signs for RSV in antenatal clinics	very low__________________________very high	☐ Not relevant
The need for research to generate more local epidemiological data on RSV	very low__________________________very high	☐ Not relevant
The need for a simple, rapid, low-cost point of care diagnostic technique	very low__________________________very high	☐ Not relevant
The need for education to raise awareness linking bronchiolitis mainly to RSV infection	very low__________________________very high	☐ Not relevant
During the RSV season, children hospitalised with RSV prevent hospitalisation of non-RSV patients	very low__________________________very high	☐ Not relevant

End of SECTION 2 and QUESTIONNAIRE

Supplementary File 3: WHO regions and countries represented by respondents in each economic group

	LD (n=18)	LM (n=31)	UM (n=21)	HIC (n=20)
Africa				
- Gambia	1	··	··	··
- Kenya	··	13	··	··
- Nigeria	··	4	··	··
- South Africa	··	··	4	··
Europe				
- Italy	··	··	··	1
- Netherlands	··	··	··	1
- Norway	··	··	··	1
- Spain	··	··	··	2
- Sweden	··	··	··	1
Eastern Mediterranean				
- Jordan	··	1	··	··
- Lebanon	··	··	1	··
- Morocco	··	1	··	··
The Americas				
- Argentina	··	··	3	··
- Brazil	··	··	10	··
- Canada	··	··	··	9
- Chile	··	··	··	1
- Colombia	··	··	1	··
- Costa Rica	··	··	2	··
South-East Asia				
- Bangladesh	8	··	··	··
- India	··	4	··	··
- Nepal	9	··	··	··
- Sri Lanka	··	6	··	··
Western Pacific				
- Australia	··	··	··	1
- Philippines	··	2	··	··
- United States	··	··	··	3

HICs: high-income countries; LD: least developed/low-income countries; LM: lower-middle-income countries; UM: upper-middle-income countries

Supplementary File 4: Top quintile of most important factors (n=20) related to the current and future management of RSV split by economic groupings*

Least Developed/Low Income		Lower Middle Income		Upper Middle Income		High Income	
**Factor**	**Loading**[†]	**Factor**	**Loading**[†]	**Factor**	**Loading**[†]	**Factor**	**Loading**[†]
The need for a simple, rapid, low-cost point-of-care diagnostic technique	2.30	The need for a simple, rapid, low-cost point-of-care diagnostic technique	2.03	The importance of a vaccine that is affordable and available to all	2.18	The importance of a vaccine that is affordable and available to all	2.45
The need for a national surveillance programme for RSV	1.90	The need for research to generate more local epidemiological data on RSV	1.79	The need for an infant vaccine for RSV	2.07	The need for a cheaper widely applicable prophylactic treatment	2.43
The need for research to generate more local epidemiological data on RSV	1.71	Access to laboratory RSV diagnostic testing is limited especially in rural areas	1.48	The need for a cheaper widely applicable prophylactic treatment	1.86	The need for a single dose long-acting prophylactic monoclonal treatment for RSV	2.24
The importance of a vaccine that is affordable and available to all	1.55	Health burden of RSV not fully recognised by the public health bodies	1.43	The high cost of prophylactic monoclonal antibody treatment, including even biosimilars	1.64	The need for an infant vaccine for RSV	2.24
The need for internationally approved, easy-to-follow, point-of-care management guidelines for RSV	1.40	Information on RSV needs to be widely available for the general population	1.38	Build on the lessons of COVID to teach parents hygiene measures for avoiding RSV infection	1.46	The high cost of prophylactic monoclonal antibody treatment, including even biosimilars	2.12
The need to establish an RSV national immunisation programme once a vaccine is available	1.36	The need for morbidity and mortality data as well as demographics of at-risk groups	1.27	The need for an antiviral or other treatment which can be used for the	1.40	The potential high cost of new palivizumab biosimilar prophylaxis products	1.94

				management of RSV			
The need for RSV point-of-care testing to be routine practice	1.36	The need to make oximeters available to all public hospitals and health facilities	1.23	The need to make oximeters available to all public hospitals and health facilities	1.38	The need to establish an RSV national immunisation programme once a vaccine is available	1.47
The need for morbidity and mortality data as well as demographics of at-risk groups	1.34	The high cost of vaccines	1.23	The need to establish an RSV national immunisation programme once a vaccine is available	1.34	The need to keep policymakers on board with evolving RSV management/prevention strategies	1.31
The paucity of local data on the prevalence and burden of RSV in the community	1.30	The need for a national surveillance programme for RSV	1.15	The high cost of vaccines	1.34	The high cost of vaccines	1.30
The need for an infant vaccine for RSV	1.27	The paucity of local data on the prevalence and burden of RSV in the community	1.12	The worry that new strategies for RSV prophylaxis and treatment will be unaffordable	1.25	Build on the lessons of COVID to teach parents hygiene measures for avoiding RSV infection	1.12
The need for education to raise awareness linking bronchiolitis to RSV infection	1.22	The need to improve the national supply chain for oxygen support especially in peripheral health centres	1.10	The importance of breast-feeding as a defence against RSV	1.25	Possibility of leveraging on the COVID experience to persuade policymakers about strategies for RSV prevention	1.10
The need for training on RSV diagnosis and enabling distinction from bacterial infection	1.20	The need for an infant vaccine for RSV	1.03	The availability of a maternal vaccine for RSV	1.23	The worry that new strategies for RSV prophylaxis and treatment will be unaffordable	1.07

The need for regular educational events, such as webinars, to update the important topic of paediatric and maternal vaccines for RSV	1.11	The need for training on RSV diagnosis and enabling distinction from bacterial infection	1.02	Understanding that RSV can be a problem in children with particular co-morbidities	1.17	The need for RSV prevention in all children not just those at high risk	1.05
The importance of international vaccine programmes such as GAVI	1.05	Average age for RSV infection is in the very young	1.00	The need for a simple, rapid, low-cost point-of-care diagnostic technique	1.14	The availability of a maternal vaccine for RSV	0.96
Health burden of RSV not fully recognised by the public health bodies	1.04	The need to keep policymakers on board with evolving RSV management/prevention strategies	0.99	The need for research to generate more local epidemiological data on RSV	1.13	Poor appreciation of the potential efficacy of combined interventions such as maternal vaccine plus monoclonal prophylaxis	0.91
The need to improve the national supply chain for oxygen support especially in peripheral health centres	1.02	The need for a cheaper widely applicable prophylactic treatment	0.98	The potential high cost of new palivizumab biosimilar prophylaxis products	1.08	The need for a national surveillance programme for RSV	0.90
Need for recognition by stakeholders that respiratory tract infections in young children are mostly viral	0.99	The need to establish an RSV national immunisation programme once a vaccine is available	0.98	Access to laboratory RSV diagnostic testing is limited especially in rural areas	1.06	Understanding that RSV can be a problem in children with particular co-morbidities	0.87
The need to understand more about co-infections with RSV since mortality may be increased	0.98	The need for internationally approved, easy-to-follow, point-of-care management guidelines for RSV	0.97	Possibility of leveraging on the COVID experience to persuade policymakers	1.00	Recognising the long-term clinical sequelae of RSV	0.85

				about strategies for RSV prevention			
The need to keep policymakers on board with evolving RSV management/prevention strategies	0.96	The need to understand more about co-infections with RSV since mortality may be increased	0.96	The need for a single dose long-acting prophylactic monoclonal treatment for RSV	0.91	The importance of timing for any new maternal vaccine for RSV during pregnancy	0.84
The need to ensure that paediatricians understand the importance of and can properly manage oxygen therapy	0.94	Lack of awareness about the morbidity and mortality associated with RSV	0.91	Involve professional associations in creating awareness of and providing advocacy for RSV	0.87	The need for morbidity and mortality data as well as demographics of at-risk groups	0.80

COVID: coronavirus; RSV: respiratory syncytial virus. *Principal component analysis that contained 86.0% of the data variance for Least Developed/Low Income countries, 91.5% for Lower Middle Income countries, 89.8% for Upper Middle Income countries, and 95.4% for High Income countries. †The loading is the relative weighting of each factor within the principal component.
